# Dietary fiber and gut bacteria shape infection susceptibility

**DOI:** 10.1038/s44320-024-00042-9

**Published:** 2024-05-23

**Authors:** Aqsa Mohammed, Robert R Jenq

**Affiliations:** Department of Genomic Medicine, The University of Texas MD Anderson Cancer Center, Houston, TX 77030 USA

**Keywords:** Microbiology, Virology & Host Pathogen Interaction

## Abstract

The specific effects of the gut microbiota on pathogen susceptibility remain unexplored. In their recent study, Desai and colleagues (Wolter et al, 2024) explore the interaction between diet, the gut microbiota and pathogen susceptibility, highlighting a diet-dependent role of *Akkermansia muciniphila*.

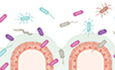

## Teaser

Gut microbiota composition has been reported to affect pathogen susceptibility but its specific effects, the underlying mechanisms and the potential influence of the diet remain unexplored. In their recent study, Desai and colleagues (Wolter et al, 2024), explore the complex interaction between diet, the gut microbiota and pathogen susceptibility, highlighting a diet-dependent role of the mucin-degrading microbe *Akkermansia muciniphila*.

The gut microbiota consists of a diverse community of microorganisms residing in the gastrointestinal tract that play crucial roles in immune response and pathogen defense. Dietary fiber is known to be beneficial for gut health as its absence has been shown to lead to significant changes in gut microbial composition and function, including disruption of the colonic mucus barrier which results in increased susceptibility to pathogenic infections (Schroeder et al, 2018) (Desai et al, 2016).

Wolter et al, used a community ecology approach to explore which bacteria affect pathogen susceptibility under conditions of fiber deprivation. They based their study on their previous work, in which they found that fiber-deprived gut microbiota increased susceptibility to *Citrobacter rodentium* (Desai et al, 2016), a rodent pathogen that models human *Escherichia coli*, and led to lethal colitis. In their latest study, the authors performed colonization of gnotobiotic mice with a strain drop-out approach, removing specific mucolytic bacteria from a 14-member synthetic microbiota (SM) community to determine the impact of different mucolytic bacteria on pathogen susceptibility in fiber-deprived diets. The 14SM community contained all four mucolytic bacteria included in the study: *Akkermansia muciniphila, Barnesiella intestinihominis, Bacteroides caccae, and Bacteroides thetaiotaomicron*. Variations of this consortium contained different species combinations e.g., the 10SM consortium contained none of the four mucolytic bacteria and 11SM contained *A. muciniphila* as the only mucolytic species.

In these analyses, following 14 days of a fiber-rich (FR) diet, half of the mice were switched to a fiber-free (FF) diet and continued this diet for 40 days. Wolter et al observed that mice on the FF diet experienced an overall expansion in the relative abundance of mucolytic bacteria, and in turn also had a reduction of fiber-degrading bacteria. In addition, FF diet was able to induce low grade inflammation as measured through concentrations of lipocalin 2 (LCN-2), particularly in 14SM mice. Notably, in FF groups with 14SM and 11SM, the only groups that included *A. muciniphila*, there was a significant reduction of propionate concentration. Propionate is a short chain fatty acid metabolite produced by *A. muciniphila* (Derrien et al, 2004), and shown to regulate *A. muciniphila’s* mucin degradation (Schwabkey et al, 2022),

To explore pathogen susceptibility, the authors infected mice with *C. rodentium*. They found that the presence of *A. muciniphila* in the 11SM condition resulted in resistance to infection in the FR condition. However, in the FF condition they observed increased *C. rodentium* levels similar to the 14SM group and increased weight loss and LCN-2 levels. This indicated that *A. muciniphila* was sufficient to exacerbate susceptibility to *C. rodentium* in a fiber-deprived diet. Analyses of immune cell populations showed significant reduction of RORγt T-helper cells in the colonic lamina propria of mice from the 14SM group on a FF diet, potentially contributing to the increased pathogen susceptibility in these mice.

Transcriptomic profiling of *C. rodentium* did not show notable effects in the presence of *A. muciniphila* in the FF diet, suggesting that pathogen susceptibility may not be mediated through direct changes in gene expression. Metatranscriptomic data analyses in infected mice showed increased expression in the FF diet for *A. muciniphila* enzymes including sialidase and β-N-acetylgalactosaminidase, which are known for targeting mucin glycans (Raba and Luis, 2023). However, the levels of these transcripts were not significantly different in the presence of *A. muciniphila* in FF mice, suggesting that other factors could be involved in modulating the mucus layer. When focusing on transcriptional changes associated with the presence or absence of *A. muciniphila*, FF mice showed increased levels of transcripts associated with anti-inflammatory properties which could potentially influence infection susceptibility.

**Figure 1:**
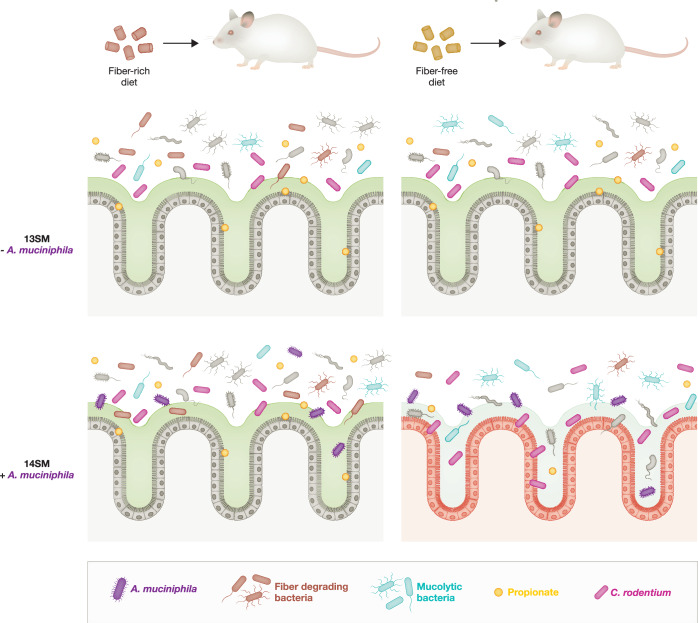
Overview of the effect of the microbiome and diet on *C. rodentium* infection susceptibility. Schematic model highlighting how diet can impact the microbiome. Mice consuming a fiber-rich (FR) diet and colonized with a synthetic gut microbiota (SM), without *A. muciniphila* (13SM) and in the presence of *A. muciniphila* (14SM) had increased the abundance of fiber-degrading bacteria and a decreased abundance of mucolytic bacteria. The opposite was observed in mice fed a fiber-free (FF) diet. 14SM ( + *A. muciniphila*) mice showed a significant difference in propionate concentrations when on an FF diet as well as increased inflammation and higher pathogen load following *C. rodentium* infection. Both diet and microbiome are shown by Wolter et al to play a role in increased susceptibility to *C. rodentium* infection, with *A. muciniphila* playing a key role.

Overall, Wolter et al demonstrated that the increased pathogen susceptibility seen in FF mice was not due to altered host immune or pathogen responses but was driven by increased mucus penetrability and changed activity of mucolytic bacteria, indicating a context-dependent interaction between diet, the microbiome and pathogen susceptibility (Fig. 1). Previous work has shown that conditions of caloric restriction, increased both the abundance of *A. muciniphila* and the expression of fucose utilization genes, fucose being a carbohydrate source of *A. muciniphila* that is a component of the glycan chains on mucin (Schwabkey et al, 2022). These studies emphasize the need for refined approaches for understanding and manipulating the gut microbiota for improved health outcomes.

The finding that diet composition can determine whether a commensal bacterium, such as *A. muciniphila*, can be harmful or beneficial is an important lesson. Effects on various microbiota members may be important to consider when designing novel therapies. The gut microbiota is highly complex and the interactions between different species as well as the impact of diet on the microbiome are complexities that need to be considered in translational studies.

This study does not fully illuminate the causal relationship between *A. muciniphila* and *C. rodentium* susceptibility, including the mechanisms by which *A. muciniphila* can change disease susceptibility depending on the diet. Future work, perhaps utilizing the potential of examining transposon mutants of *A. muciniphila* (Davey et al, 2023) in strain drop-out experiments, could be informative in that respect. Finally, there is potential for quantifying and characterizing *A. muciniphila* as a biomarker to predict pathogen susceptibility. This opens the door for future studies to identify microbiome-based biomarkers and to advance our understanding of how diet can modify the behavior of microbes in the context of different diseases.
